# Exogenous erythropoietin increases hematological status, fat oxidation, and aerobic performance in males following prolonged strenuous training

**DOI:** 10.14814/phy2.16038

**Published:** 2024-05-16

**Authors:** Devin J. Drummer, Julie L. McNiff, Emily E. Howard, Jess A. Gwin, Christopher T. Carrigan, Nancy E. Murphy, Marques A. Wilson, Julia Michalak, Benjamin J. Ryan, James P. McClung, Stefan M. Pasiakos, Lee M. Margolis

**Affiliations:** ^1^ Military Nutrition Division U.S. Army Research Institute of Environmental Medicine Natick Massachusetts USA; ^2^ Oak Ridge Institute for Science and Education Belcamp Maryland USA; ^3^ Combat Feeding Division U.S. Army Combat Capabilities Development Command (DEVCOM) Natick Massachusetts USA; ^4^ Thermal and Mountain Medicine Division U.S. Army Research Institute of Environmental Medicine Natick Massachusetts USA; ^5^ Office of Dietary Supplements, National Institutes of Health U.S. Department of Health and Human Services Bethesda Maryland USA

**Keywords:** hematocrit, hemoglobin, mitochondria, substrate oxidation, V̇O_2_peak

## Abstract

This study investigated the effects of EPO on hemoglobin (Hgb) and hematocrit (Hct), time trial (TT) performance, substrate oxidation, and skeletal muscle phenotype throughout 28 days of strenuous exercise. Eight males completed this longitudinal controlled exercise and feeding study using EPO (50 IU/kg body mass) 3×/week for 28 days. Hgb, Hct, and TT performance were assessed PRE and on Days 7, 14, 21, and 27 of EPO. Rested/fasted muscle obtained PRE and POST EPO were analyzed for gene expression, protein signaling, fiber type, and capillarization. Substrate oxidation and glucose turnover were assessed during 90‐min of treadmill load carriage (LC; 30% body mass; 55 ± 5% V̇O_2_peak) exercise using indirect calorimetry, and 6‐6‐[^2^H_2_]‐glucose PRE and POST. Hgb and Hct increased, and TT performance improved on Days 21 and 27 compared to PRE (*p* < 0.05). Energy expenditure, fat oxidation, and metabolic clearance rate during LC increased (*p* < 0.05) from PRE to POST. Myofiber type, protein markers of mitochondrial biogenesis, and capillarization were unchanged PRE to POST. Transcriptional regulation of mitochondrial activity and fat metabolism increased from PRE to POST (*p* < 0.05). These data indicate EPO administration during 28 days of strenuous exercise can enhance aerobic performance through improved oxygen carrying capacity, whole‐body and skeletal muscle fat metabolism.

## 1 INTRODUCTION

Chronic, strenuous physical activity can result in overreaching and negative hematological adaptations leading to reduced oxygen carrying capacity in the blood (Bellinger et al., 2020; Cheng et al., 2020; Hoier et al., 2013; McClung et al., 2013; McClung, Karl, Cable, Williams, Young, et al., 2009; Merkel et al., 2009; Pasiakos et al., 2016). Prolonged periods of overreaching may be unavoidable such as during strenuous military operations. Prolonged strenuous military operations decrease hemoglobin (Hgb) and hematocrit (Hct) (McClung et al., 2013; McClung, Karl, Cable, Williams, Young, et al., 2009; Merkel et al., 2009; Pasiakos et al., 2016). These negative hematological adaptations have been linked to increased circulating concentrations of IL‐6 (Hennigar et al., 2017) and hepcidin (Kemna et al., 2005; Nemeth et al., 2004) which reduces absorption and alters the distribution of iron throughout the body (Kong et al., 2014). Reduced oxygen carrying capacity has been associated with declines in physical performance (McClung, Karl, Cable, Williams, Nindl, et al., 2009; McClung, Karl, Cable, Williams, Young, et al., 2009). Consuming daily iron supplements can partially alleviate declines in Hgb and Hct during strenuous training (McClung, Karl, Cable, Williams, Nindl, et al., 2009; Pompano & Haas, 2017); however, values do not fully recover to the levels observed when iron supplements are consumed without physical training (Pompano & Haas, 2017). As such, more robust interventions may be warranted to mitigate negative hematological adaptations and declines in physical performance associated with extended periods of strenuous activity.

Exogenous erythropoietin (EPO) is one potential intervention that could lessen negative hematological adaptations that result from prolonged physically demanding military operations. EPO is produced in the kidneys and binds EPO receptors on the surface of bone marrow derived red blood cell precursors (Elliott et al., 2008; Lundby & Olsen, 2011). The stimulation of these hematopoietic progenitors by endogenous EPO prompts their proliferation and differentiation (erythropoiesis) into red blood cells, thus increasing Hgb and Hct (Elliott et al., 2008; Lundby & Olsen, 2011). Exogenous EPO also results in higher levels of Hgb and Hct compared to placebo (Caillaud et al., 2015; Durussel et al., 2013; Thomsen et al., 2007), but the response may be limited without adequate iron (Tarng et al., 1999). Our laboratory has recently demonstrated that secondary elevations in circulating EPO following exogenous testosterone administration compared to placebo resulted in lower hepcidin and IL‐6, increased iron availability, and Hgb and Hct following 21 days of exercise‐induced (~1400 kcal/day) energy deficits (Hennigar, Berryman, et al., 2020). Furthermore, exogenous EPO may also stimulate non‐hematological adaptations independent of increased red blood cell volume and V̇O_2_max that enhance physical performance (Annaheim et al., 2016). Non‐hematological adaptations may occur in skeletal muscle, increasing markers of mitochondrial biogenesis (Wang et al., 2013) and improving mitochondrial respiratory capacity (Plenge et al., 2012). However, the effectiveness of exogenous EPO on hematological and non‐hematological adaptations during a period of high physical demand is unknown.

The primary objective of this study was to determine the effect of EPO on hematological adaptations and aerobic performance during a period of prolonged, strenuous exercise. The secondary objective was to assess the impact of EPO during strenuous exercise on non‐hematological adaptations such as substrate oxidation, glucose turnover, skeletal muscle gene expression, skeletal muscle signaling, and skeletal muscle fiber type and capillarization. We hypothesized that EPO would maintain Hgb, Hct, and aerobic performance during 28 days of strenuous exercise. Additionally, we hypothesized that EPO would increase fat oxidation and decrease carbohydrate oxidation during steady‐state aerobic exercise. We also hypothesized that shifts in substrate oxidation would reflect an increased oxidative phenotype and capillarization in skeletal muscle following 28 days of strenuous exercise compared to baseline assessments.

## 2 METHODS

### 2.1 Participants

Eight healthy males (age: 18–39 years) completed this longitudinal study at the U.S. Army Research Institute of Environmental Medicine. Eligible participants were males or females who recreationally active (2–4 days per week of aerobic and/or resistance exercise) and weight stable (±5lbs) for at least 2 months prior to the study beginning. All participants were US Army service members and free from acute or chronic disease (e.g., cardiovascular, pulmonary, and metabolic disease), food allergies, and medication allergies. Participants were required to refrain from consuming alcohol or nicotine products for the duration of the study. Each participant provided written informed consent prior to beginning data collection. All study procedures were approved by the U.S. Army Medical Research and Development Command (Fort Detrick, Fredericksburg, MD, USA) Institutional Review Board (IRB) and are in accordance with the Declaration of Helsinki. The study was registered with clinicaltrials.gov (NCT05078138).

### 2.2 Study design

This longitudinal study consisted of a baseline phase, followed by a 28‐day EPO injection phase (Figure S1). Assessments of body composition, resting metabolic rate (RMR), V̇O_2_peak, aerobic performance (5‐km time trial; TT), substrate oxidation, glucose turnover, and muscle fiber type and capillarization were completed during the baseline phase (PRE). Participants then received EPO injections three times per week for 28 days. During the EPO phase, blood samples were collected weekly to monitor changes in Hgb and Hct. Participants completed a TT every 7 days during the injection phase to determine time course change in performance with EPO compared to baseline. Exercise training was controlled during the EPO phase, consisting of a prescribed combination of endurance‐ and resistance‐type exercise. V̇O_2_peak was reassessed after 14 days of EPO injection to adjust exercise intensities as appropriate to account for changes in V̇O_2_peak. At the conclusion (POST) of the EPO phase, body composition, V̇O_2_peak, substrate oxidation, glucose turnover, muscle gene expression, protein signaling, and muscle fiber type and capillarization were reassessed. All food and beverages (except water) were provided to participants for the duration of the study.

### 2.3 Anthropometrics

Height was measured to the nearest 0.1 cm using a stadiometer at baseline. Body mass was measured after an overnight fast (≥8 h) using a calibrated digital scale to the nearest 0.1 kg at baseline and Days 7, 14, 21, and 28 during the EPO phase to confirm weight maintenance. Body composition was determined PRE and POST to characterize changes in fat mass and fat‐free mass using dual energy x‐ray absorptiometry (DEXA, DPX‐IQ, GE Lunar Corporation, Madison, WI, USA).

### 2.4 Resting metabolic rate

Following a 10‐h overnight fast, RMR was measured using open circuit indirect calorimetry (True Max 2400, Parvomedics, Sandy, Utah, USA) at baseline. Participants rested in the supine position for approximately 30‐min before measurement in a quiet and dim, temperature regulated room. To minimize error, participants were instructed to restrict movement once the hood was placed over their heads to collect expired air. The test was discontinued when 20‐min of steady‐state oxygen consumption (V̇O_2_) and carbon dioxide production (V̇CO_2_) were recorded. The RMR, multiplied by a factor of daily living (1.3 for sedentary behavior) was added to exercise‐induced energy expenditures to determine the energy needs for study diets.

### 2.5 Study diets

Registered dietitians developed individualized daily menus using Food Processor SQL (ESHA Research, Salem, OR, USA; Version 10.14). The diets were derived primarily from components of the US military Meals Ready‐to‐Eat (MRE) rations to achieve appropriate macronutrient proportions. Energy content of diets was adjusted during prescribed exercise, TT, and substrate oxidation days to match participants estimated energy needs to maintain body weight throughout the study. Diets provided approximately 55% carbohydrate, 15% protein, and 30% fat. Dietary iron was controlled, providing a minimum of 18 mg/day to ensure that the recommended dietary allowance was met regardless of biological sex.

### 2.6 Erythropoietin injections

Recombinant human EPO (PROCRIT, EPOETIN ALFA, Janssen Products, LP, Titusville, NJ, USA) was injected subcutaneously three times per week for 4 weeks at a dose of 50 IU/kg body mass. This dose and duration have previously been used to elicit hematological adaptations and increase physical performance, without adverse events (Caillaud et al., 2015; Connes et al., 2003; Thomsen et al., 2007). As described earlier, Hgb and Hct were assessed weekly for safety monitoring. Each were measured in whole blood using the i‐STAT1 (Abbott Point of Care Diagnostics, Princeton, NJ; cat:03P75‐07) and associated CHEM8+ cartridges (Abbott Point of Care Diagnostics, Princeton, NJ; cat:09P31‐26). EPO injections were halted if Hct increased above 50% or Hgb increased more than 1 g/dL over 1‐week. If needed, Hct and Hgb were reassessed 3 days after halting EPO injections. No volunteer reached a Hct of ≥50% throughout this intervention. Two volunteers missed one injection each due to their Hgb rising >1 g/dL over a week. EPO injections resumed after Hgb levels stabilized 3 days following the halted injection. Before starting the EPO phase, participants completed a short clinically relevant health questionnaire and had vital signs assessed (blood pressure, heart rate, and SpO_2_). Participants were monitored for 2 weeks after the final injection, with weekly safety blood sample collections and daily monitoring of vital signs and health questionnaires.

### 2.7 Prescribed exercise

During the EPO phase, participants performed prescribed exercise training for 4 days per week and had 1 day of weekly physical performance testing (TT). Participants performed both weighted (treadmill or outdoor; walking, running, and load carriage) and unweighted (cycle ergometry) endurance‐type exercise, and resistance‐type exercise (3 transitioning to 4 sets of 10 repetitions at body weight, progressing to weighted squats, and lunges) throughout the protocol each day. Exercise was designed to elicit energy expenditures of 1200–1500 kcal per day (First 2 weeks were ~1200 kcal/day progressing to ~1500 kcal/day during the final 2 weeks). Prescribed endurance‐type exercise was low‐to‐high intensity (30%–85% V̇O_2_peak) using the American College of Sports Medicine metabolic equations for steady‐state exercise (Glass & Gregory, 2007) and the compendium of metabolic equivalents for physical activities to prescribed the necessary volume to elicit the desired caloric expenditure (1200 kcal/day Week 1 and 2, or 1500 kcal/day Week 3 and 4). This level of training is similar to previously conducted arduous military training in garrison (Barringer et al., 2018). Exercise intensity was based on V̇O_2_peak assessed prior to the onset of study exercise and readjusted 2 weeks into the EPO phase.

### 2.8 V̇O_2_peak

Following an overnight fast (10 h), participants completed a maximal aerobic exercise session to determine peak oxygen uptake (V̇O_2_peak) on a treadmill. V̇O_2_peak was determined using an indirect, open circuit respirator system (Parvomedics) at PRE, Days 14, and 26 of the injection phase. Participants were given adequate time to become familiar with the testing procedures and allowed a 3‐min self‐paced warm‐up on the treadmill. The participants began by running for 4 min at a pace predetermined as comfortable at a 0% grade. At 4‐min, the grade was increased to 4% followed by an additional 2% every 2 min thereafter until volitional exhaustion. Changes in V̇O_2_peak during EPO phase were used to adjust daily exercise prescription, and the substrate oxidation test at POST.

### 2.9 Time trial

Following a 10‐h overnight fast, participants completed a 5‐km TT. The treadmill was set at a constant 1% grade (Jones & Doust, 1996) for the entire test. Following a warm‐up period, the treadmill was set to 5 mph, then participants adjusted the treadmill speed to complete the distance as quickly as possible. Participants were blinded to the treadmill speed. The only feedback given was distance covered at half mile increments. Heart rate was monitored throughout the TT and recorded at half mile increments along with rate of perceived exertion. No motivation was provided during the TT. Participants were allowed to consume water ad libitum. Following completion of the test, a self‐selected cool‐down occurred. Participants completed a minimum of two practice exercise sessions to ensure they were familiar with the performance test prior to the first official TT.

### 2.10 Substrate oxidation test

Following a 10‐h overnight fast, a catheter was placed in the antecubital vein of each arm, one for stable isotope (6,6‐[^2^H_2_] glucose tracer) infusion and one for sampling blood. Following an initial blood sample collection to determine background enrichments, a primed, continuous infusion of 6,6‐[^2^H_2_] glucose began (prime, 82.2 μmol∙kg^−1^; continuous rate, 0.78 μmol∙kg^−1^∙min^−1^, Figure S2) for 100 min before and throughout the 90‐min bout of aerobic exercise. Immediately before exercise, a percutaneous muscle biopsy was obtained from the vastus lateralis using a 5 mm Bergstrom needle with manual suction while the participant was under local anesthesia (1% lidocaine). Participants then performed 90 min of steady‐state (~55 ± 5% of V̇O_2_peak) load carriage exercise with a weighted vest (~30% body mass) on the treadmill at PRE and POST. The speed and grade of the treadmill were determined using the ACSM metabolic equation for walking based on desired V̇O_2_ (American College of Sports Medicine, 2006). Prior to the substrate oxidation test day, participants performed a practice session of the treadmill exercise to confirm that the prescribed speed and grade were appropriate to induce the target V̇O_2_. During exercise, V̇O_2_, V̇CO_2_, and HR were measured six times at approximately 0, 20, 40, 60, 70, and 80 min. Energy expenditure, carbohydrate, and fat oxidation rates were calculated from V̇O_2_ (L/min) and V̇CO_2_ (L/min) during the 90‐min exercise bout as described by Jeukendrup and Wallis (2005; Péronnet & Massicotte, 1991):

$$\begin{array}{cc} \text{Energy expenditure}\;\left(\text{kcal}/\min\right)= & \left(0.575\times \dot{V}{\mathrm{CO}}_{2}\right)+\\ & \left(4.435\times \dot{V}O_{2}\right). \end{array}$$

$$\begin{array}{cc} \mathrm{Fat}\;\text{oxidation}\;\left(g/\min\right)= & 1.695\times \dot{V}O_{2}\;\left(L/\min\right)-1.701\times \\ & \dot{V}{\mathrm{CO}}_{2}\;\left(L/\min\right). \end{array}$$

$$\begin{array}{c} \text{Total carbohydrate oxidation}\;\left(g/\min\right)\\ =4.585\times \dot{V}{\mathrm{CO}}_{2}\;\left(L/\min\right)-3.226\times \dot{V}O_{2}\;\left(L/\min\right). \end{array}$$

### 2.11 Blood samples

Blood sampling occurred after a 10‐h overnight fast. Blood collections assessing Hgb and Hct for safety were conducted using venipuncture. During the substrate oxidation tests, blood was collected using an indwelling catheter kept patent using IV saline at approximately −100, −20, 0, 20, 40, 60, and 80 min for assessment of glucose isotope enrichments (6,6‐[^2^H_2_] glucose, Cambridge Isotope Laboratories, Andover, MA; cat: DLM‐349‐MPT‐PK). Blood collected at −100 and 80 min was also used to assess IL‐6 and hepcidin concentrations (R&D Systems, Inc, Minneapolis, MN; cat: D6050 and DRG International, Springfield, NJ; cat: EIA‐5782 respectively). Tubes yielding serum were allowed to clot at room temperature (RT) for at least 10 min then centrifuged at 3600 rpm for 10 min at 4°C. Blood was then stored at −80°C until analyzed.

### 2.12 Plasma glucose turnover

For glucose kinetic analysis, the tracer/trace ratio (6,6‐[^2^H_2_] glucose/glucose) of blood samples was measured on the pentaacetate derivative by gas‐chromatography‐mass spectrometry (Metabolic Solutions, Inc., Nashua, NH, USA). Specifically, the calculation of plasma glucose turnover was determined using the Steele equation with modifications for non‐steady state (Wolfe & Chinkes, 2005). Enrichment (E) was expressed as mole percent excess (MPE); calculated as (TTR)/(1 + TTR), where TTR was the tracer to tracee ratio. Appropriate corrections for skewed abundance distribution and overlapping spectra were made for the TTR of the glucose tracer, 6,6‐[^2^H_2_] glucose (Wolfe & Chinkes, 2005).

$$\text{Glucose}\;R_{a}\;\left(\text{Total}\;R_{a}\right)=\left(F–\left(\left(pV\times \left(\left(C_{2}+C_{1}\right)\right)/2\right)\times \left(\left(E_{2}–E_{1}\right)/\left(t_{2}–t_{1}\right)\right)\right)\right)/\left(\left(E_{2}+E_{1}\right)/2\right).$$

$$\text{Glucose}\;R_{d}=\text{Total}\;R_{a}–\left(pV\;\left(C_{2}–C_{1}\right)/\left(t_{2}–t_{1}\right)\right).$$

$$\begin{array}{c} \text{Metabolic Clearance Rate}\;\left(\mathrm{MCR}\right)\\ =\text{Glucose}\;R_{d}/\left(\left(C_{2}+C_{1}\right)/2\right). \end{array}$$

Where *F* represents the infusion rate of 6,6‐[^2^H_2_] glucose; *p*V is the effective volume of distribution for glucose, *C*_1_ and *C*_2_ are plasma glucose concentrations at *t*_1_ and *t*_2_, respectively, *E*_1_ and *E*_2_ are plasma enrichments of 6,6‐[^2^H_2_] glucose at *t*_1_ and *t*_2_, respectively.

### 2.13 Immunohistochemistry

Vastus lateralis muscle was collected in a rested and fasted state PRE and POST the EPO phase. A portion of the muscle collected was assessed for myofiber type distribution and skeletal muscle capillary alterations as previously described (Kelly et al., 2018; Murach et al., 2019). Briefly ~50 mg of skeletal muscle was removed for histological processing from the total muscle collected. Fibers were aligned, placed in a mount consisting of a mixture of Tissue Tek O.C.T compound (Sakura Finetek, Torrance, CA) and Tragacanth gum (Sigma‐Aldrich, USA) and frozen in liquid nitrogen (LN_2_) cooled isopentane. All samples were stored at −80°C until ready for further processing.

Mounts were cut at 6 μm, dried for 30 min then stored at −20°C until ready for immunostaining. For each stain, sections were thawed, fixed, and then blocked in 5% goat serum. Sections were incubated in components from the following primary and secondary list depending on the stain: MHCI antibody (1:50 in 1% goat serum, BA‐D5, Developmental Studies Hybridoma Bank [DSHB], University of Iowa) MHCIIa antibody (final concentration of 1.5 μg/mL, A4.74, DSHB, University of Iowa), laminin (laminin Beta2/Gamma (A5), MA1‐06100, Thermo Fisher Scientific, USA), FITC conjugated UEA I (1:100 in 1% goat serum, Vector Laboratories, Newark, CA, cat: FL‐1061), 594 GAM IgG2b (1:400 in 1% goat serum, Thermo Fisher Scientific, Waltham, MA, cat: A‐21145), 488 GAM IgG1 (1:400 in 1% goat serum, Thermo Fisher Scientific, Waltham, MA, cat: A‐21121), and 647 GAR IgG (H + L) (1:200 in 1% goat serum, Thermo Fisher Scientific, Waltham, MA, cat: A‐21247). Sections were covered with Vector Shield with DAPI (Thermo Fisher Scientific, Waltham, MA, cat: NC1695563) and stored at −20°C in a horizontal position until ready for imaging.

All images were captured at 20X using Nikon Eclipse Ti2 Confocal. Myofiber type, capillary counts by fiber type, and capillary density by area were determined using NIS Elements AR Software version 5.40.01 64 bit Microscope (Nikon, NY, USA). For fiber type analyses, type I and IIa fibers were defined by the sole staining of the respective antibodies (BA‐D5, and A4.74). Hybrid fibers IIX/IIAX were defined as the combination A4.74 and negative staining, while I/IIA hybrid fibers were defined as a combination of BA‐D5 and A4.74. All interpretations were made by the same analyst and a representative image is shown in Figure 5a.

### 2.14 Gene expression

RNA from approximately 20 mg of skeletal muscle was extracted using the miRNeasy Mini Kit (Qiagen, Valencia, CA, USA, cat 217004) using standard procedures. RNA quality and concentration was assessed using Nanodrop ND‐2000 spectrophotometer (Nanodrop, Wilmington, DE, USA). Equal amounts of total RNA (500 ng) were reverse transcribed using the high‐capacity cDNA reverse transcription kit (Thermo Fisher Scientific, USA, cat 4368814). Gene expression analyses were prepared with TaqMan Fast Advanced Master Mix (Thermo Fisher Scientific, USA, cat 4444556) and probed with commercially available TaqMan probes (TATA‐box binding protein, TBP, Hs00427620_m1; glyceraldehyde 3‐phospate dehydrogenase, GAPDH, Hs02786624_g1; mitochondrial transcription factor A, TFAM, Hs01082775_m1; peroxisome proliferator‐activated receptor gamma coactivator 1‐alpha, PPARGC1, Hs00173304_m1; fatty acid binding protein 3 FABP3, Hs07287863_m1; cytochrome c oxidase four, COX4, Hs00971639_m1; vascular endothelia growth factor A, VEGFA, Hs00900055_m1; cluster of differentiation 36, CD36, Hs00354519_m1; hydroxyacyl‐CoA dehydrogenase trifunctional multienzyme complex subunit alpha, HADHA, Hs00426191_m1; carnitine palmitoyltransferase I, CPT1A, Hs00912671_m1; fatty acid synthase, FASN, Hs001005622_m1; and acetyl‐CoA carboxylase alpha, ACACA, Hs01046047_m1). Gene expression was determined using the QuantStudio 6 Flex Real‐Time PCR System (Applied Biosystems, Thermo Fisher Scientific, USA). Gene expression was normalized to the geometric mean of TBP and GAPDH. Fold changes were calculated using the ΔΔ cycle threshold (ΔΔCT) method, with POST expressed relative to PRE. All gene expression data were log transformed for analysis.

### 2.15 Protein signaling

Protein homogenates were made from approximately 20 mg of skeletal muscle and centrifuged for 15 min at 12,000 times gravity (4°C). Lysate was collected and protein concentrations were determined using 660 assays (Thermo Scientific, ref 22660). Running samples were solubilized in Laemmli buffer and denatured at 95۠°C for 5 min. Equal amounts of protein (15 μg) were loaded per lane and separated by SDS‐PAGE using precast Tris–HCL gels (Bio‐Rad, Hercules, CA, USA, cat 5671095). Following separation, proteins were transferred to PVDF membranes, blocked with fat free milk and probed with commercially available primary antibodies (heat shock protein 90, HSP90 (housekeeping protein); and cytochrome c oxidase four, COXIV; Cell Signaling Technology, Beverly, MA, USA, cat numbers: 4877S and 4850S respectively) overnight with agitation at 4°C. Secondary (Cell Signaling Technology, Beverly, MA, USA, cat 7074P2) and chemiluminescent reagents were applied (HSP90: Super Signal™, West Pico Kit; Thermo Fisher Scientific, USA, cat 34580, or COXIV Super Signal™, West Femto Maximum Sensitivity Substrate, Thermo Fisher Scientific, USA, cat 34095) then images were quantified using a ChemiDoc XRS (Bio‐Rad, Hercules, CA, USA) and accompanied software. HSP90 was deemed as an appropriate housekeeping protein for these experiments as it was not different from PRE to POST (*p* ≥ 0.05). Blot images with the target protein and the molecular weight where membranes were cut for all participants can be found in Figure S3.

### 2.16 Statistical analysis

A priori sample size and power calculations were determined based on multiple outcomes within this work (time to exhaustion, Hct, and fat oxidation). A sample size of eight was deemed necessary based on an effect size of >1 in previously published works (Caillaud et al., 2015; Plenge et al., 2012; Thomsen et al., 2007). Statistical analyses were conducted using SPSS (IBM Corp. Armonk, NY), or GraphPad Prism 10.1.1. Shapiro–Wilk tests were used to determine normality of data. Paired *t*‐tests were used to assess muscle gene expression and protein signaling, immunohistochemistry, serum EPO concentrations, energy expenditure, and delta (∆; load carriage: 80 minus ‐20‐min) IL‐6. Delta (∆; load carriage: 80 minus ‐20‐min) hepcidin PRE versus POST was non‐normally distributed so a Wilcoxon matched‐pairs sign rank test was used. Mixed‐model ANOVA, with participant as a random variable, was used to assess main effects of time for TT performance, V̇O_2_peak, Hgb, and Hct. Mixed‐model ANOVA was used to assess main effects of phase (PRE vs. POST), time, and their interaction for glucose turnover and substrate oxidation. The alpha level was Bonferroni adjusted for multiple comparisons, with the level for statistical significance set at *p* < 0.05. All data are presented as mean ± SD.

## 3 RESULTS

### 3.1 Participant anthropometrics, exercise‐induced energy expenditures, and dietary intake

Exercise‐induced energy expenditures were 1215 ± 56 kcal/day for 4 days per week for the first 2 weeks of the EPO phase, and 1453 ± 56 kcal/day for 4 days per week for the second 2 weeks. Average daily dietary intake throughout the EPO phase was 3162 ± 265 kcal/day; 5.6 ± 0.2 g carbohydrate/kg/day; 1.6 ± 0.1 g protein/kg/day; 1.3 ± 0.1 g fat/kg/day; and 39.5 ± 4.4 mg iron/day. Specific intakes for each study day (i.e., exercise, substrate oxidation test, TT, or rest) appear in Table S1. There was no change in body mass, BMI, fat mass, and fat free mass from PRE to POST (Table 1).

**TABLE 1:** Participant and steady‐state exercise characteristics.

	PRE	POST	*p* value
Age (years)	20 ± 3		
Height (m)	1.77 ± 0.03		
Weight (kg)	78.91 ± 8.68	78.54 ± 9.02	0.85
BMI (kg/m^2^)	25.17 ± 3.10	25.05 ± 3.17	0.81
Fat mass (kg)	18.07 ± 4.15	17.61 ± 4.48	0.32
Fat free mass (kg)	60.60 ± 5.37	60.92 ± 5.43	0.49
Treadmill speed (mph)	3.33 ± 0.09	3.36 ± 0.05	0.20
Treadmill grade (%)	4.73 ± 0.84	6.29 ± 0.83	**0.001** *
Average VO_2_ (L/min)	1.89 ± 0.17	2.09 ± 0.14	**0.001** *
Average VCO_2_ (L/min)	1.62 ± 0.14	1.77 ± 0.12	**0.03** *
Relative exercise intensity (% VO_2peak_)	54.46 ± 2.15	54.52 ± 2.25	0.93
Average RER (VCO_2_/VO_2_)	0.86 ± 0.04	0.84 ± 0.02	0.52
Energy expenditure (kcals/min)	9.34 ± 0.82	10.31 ± 0.71	**0.002** *

*Note*: Mean ± SD; *n* = 8. The bolded *p* values indicates, variables which were significantly different.

* *p <* 0.05.

### 3.2 Blood analytes

**FIGURE 1:**
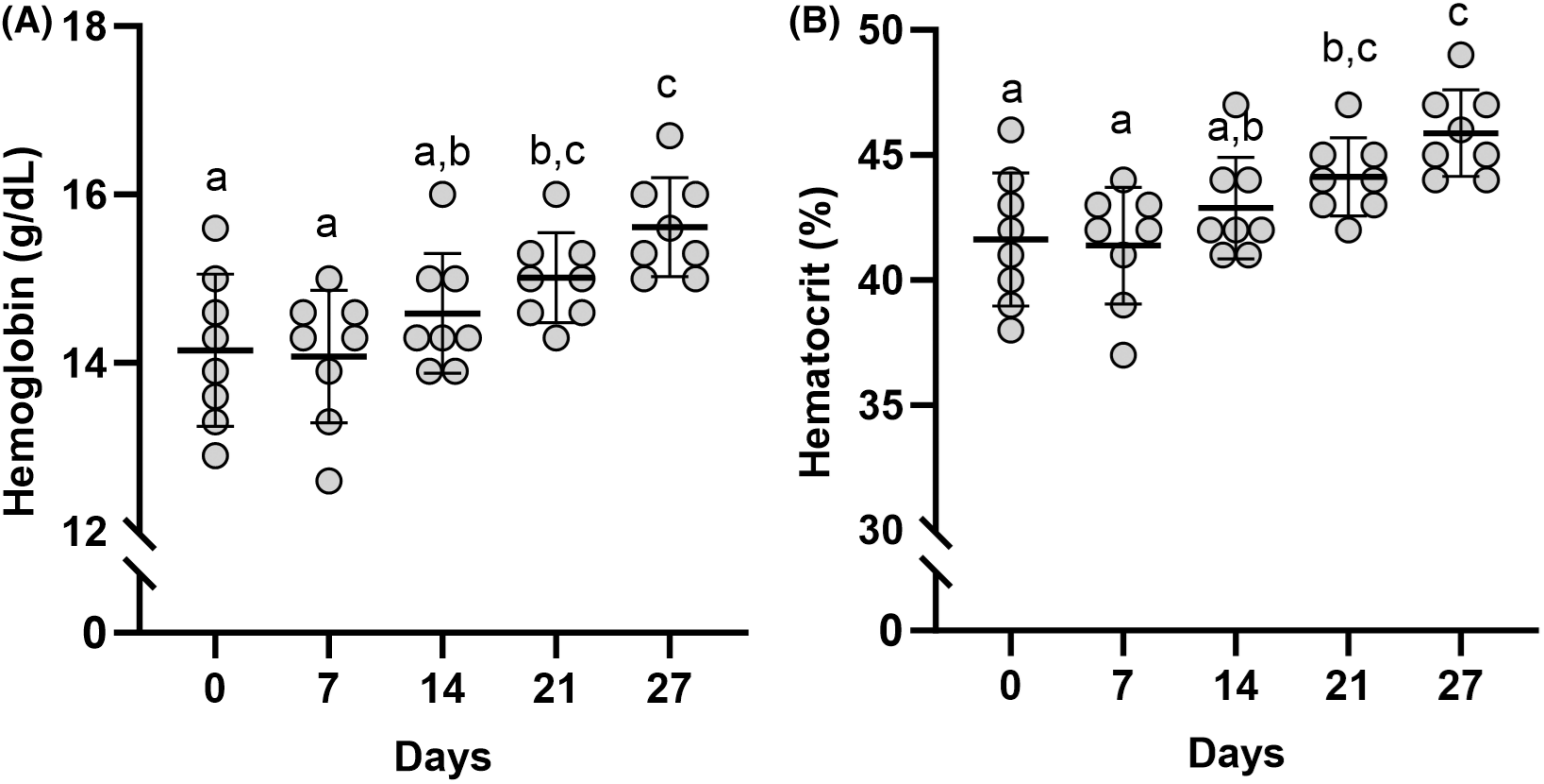
Hematological adaptations to training and erythropoietin administration. (A) Hemoglobin (g/dL) assessed throughout the 28‐day intervention. (B) Hematocrit (%) assessed throughout the 28‐day intervention. Data represented as mean ± SD. Timepoints not sharing letters are different than each other. *n* = 8 males. Statistical test = mixed model ANOVA. Significance determined as *p <* 0.05.

**FIGURE 2:**
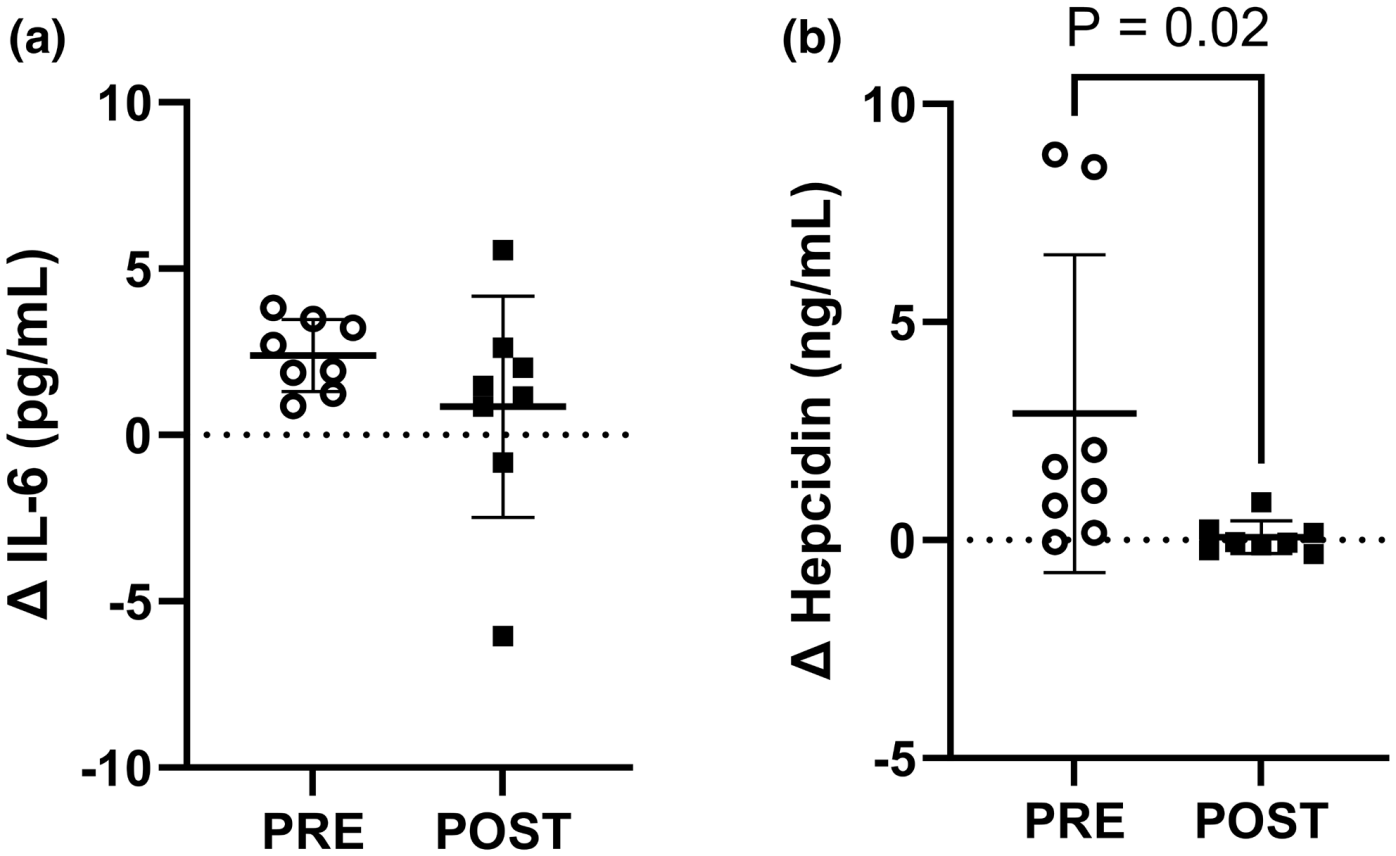
Serum analytes in response to training and erythropoietin (EPO) administration. (a) Change in Interleukin‐6 (IL‐6, pg/mL) assessed prior to and 80 min into load carriage exercise at PRE and POST timepoints. (b) Hepcidin (ng/mL) assessed prior to and 80 min into load carriage exercise at PRE and POST timepoints. Dotted line at 0 represents the blood draw prior to load carriage. Data represented as mean ± SD. *n* = 8 males. Statistical test = A: paired *t*‐test, B: Wilcoxon matched‐pairs sign rank test.

Serum EPO concentrations increased (*p* < 0.001) from PRE (8.27 ± 1.72 mIU/m) to POST (21.91 ± 5.34 mIU/m). Hgb and Hct were unchanged from PRE on Days 7 and 14, but increased (*p* < 0.001) by 0.86 ± 0.65 g/dL and 2.50 ± 2.00%, respectively, on day 21, and 1.46 ± 0.71 g/dL and 4.25 ± 2.19%, respectively, on Day 27 (Figure 1A,B). There was no difference (*p* ≥ 0.05) in ∆IL‐6 during load carriage exercise at POST compared with PRE (Figure 2a). In response to acute load carriage exercise, ∆hepcidin decreased (*p* = 0.02) POST compared with PRE (Figure 2b).

### 3.3 V̇O_2_peak and time trial performance

**FIGURE 3:**
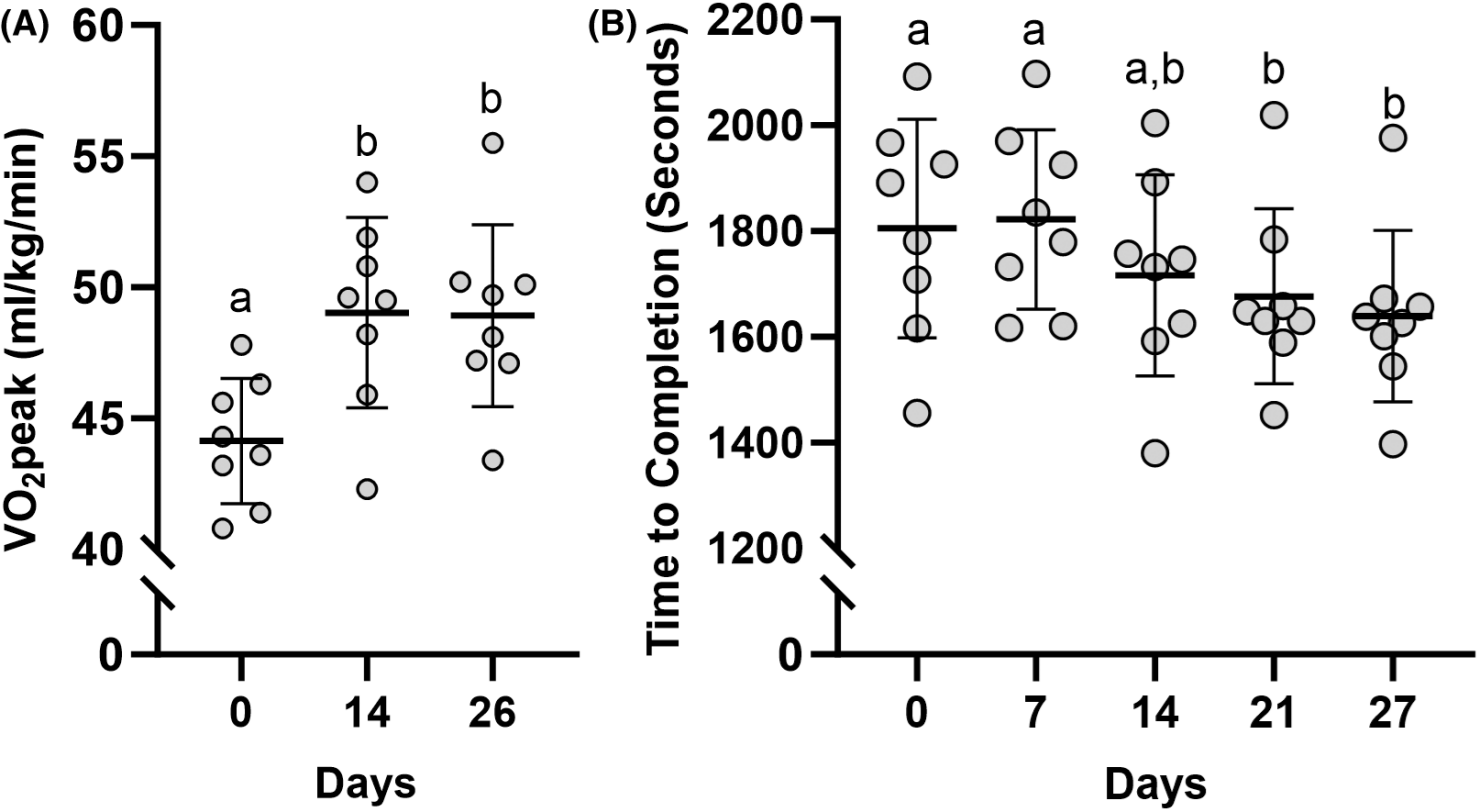
Performance adaptations to training and erythropoietin administration. (A) Treadmill V̇O_2_peak (mL/kg/min) assessed throughout the 28‐day intervention. (B) Five‐km treadmill time to completion (s) assessed throughout the 28‐day intervention. Individual data points represented as circles. Data represented as mean ± SD. Timepoints not sharing letters are different than each other. *n* = 8 males. Statistical test = mixed model ANOVA. Significance determined as *p <* 0.05.

V̇O_2_peak increased (*p* < 0.001) 4.90 ± 2.96 and 4.79 ± 2.05 mL/kg/min on Days 14 and 26, respectively from PRE (Figure 3A). Time trial performance on Days 7 and 14 were not different from PRE, but time to complete the 5 km run improved (*p* < 0.001) 128 ± 113 s on Day 21 and 166 ± 143 s on Day 27 relative to PRE (Figure 3B).

### 3.4 Substrate oxidation and glucose turnover

**FIGURE 4:**
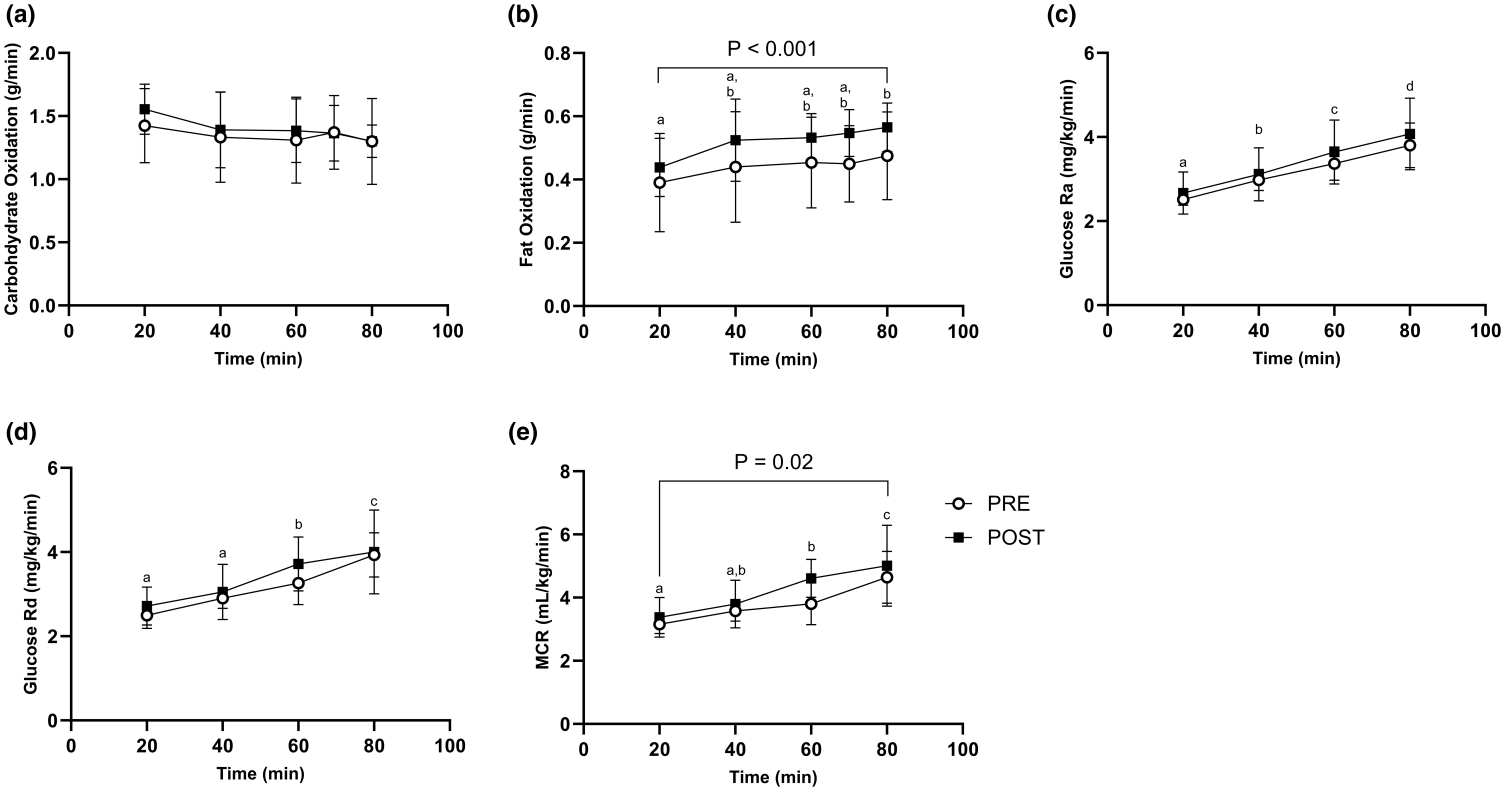
Substrate dynamics in response to training and erythropoietin administration. (A) Carbohydrate Oxidation (g/min) during the load carriage exercise PRE and POST. (B) Fat Oxidation (g/min) during the load carriage exercise PRE and POST. (C) Glucose rate of appearance (mg/kg/min) during the load carriage exercise PRE and POST. (D) Glucose rate of disappearance (mg/kg/min) during the load carriage exercise PRE and POST. (E) Metabolic Clearance Rate (mL/kg/min) during the load carriage exercise PRE and POST. Data represented as mean ± SD. *n* = 8 males. Timepoints not sharing letters are different than each other; *p <* 0.05. Numeric *p* value = different than PRE. Statistical test = mixed model ANOVA.

Treadmill grade, V̇O_2_, and V̇CO_2_ were increased (*p* < 0.05) and relative exercise intensity was unchanged from PRE to POST (Table 1). Energy expenditure increased (*p* = 0.002) during load carriage exercise from PRE to POST (Table 1). Carbohydrate oxidation was no different at POST compared with PRE during load carriage exercise (Figure 4A). Fat oxidation increased (*p* < 0.001) during load carriage exercise from PRE to POST (Figure 4B). Glucose R_a_ and R_d_ increased (*p* < 0.05) during load carriage exercise, regardless of phase (Figure 4C,D). Independent of time point, MCR increased (*p* = 0.02) at POST compared with PRE during load carriage exercise (Figure 4E). MCR increased (*p* < 0.001) over time during load carriage exercise, regardless of phase.

### 3.5 Impact of EPO and strenuous exercise on skeletal muscle

**FIGURE 5:**
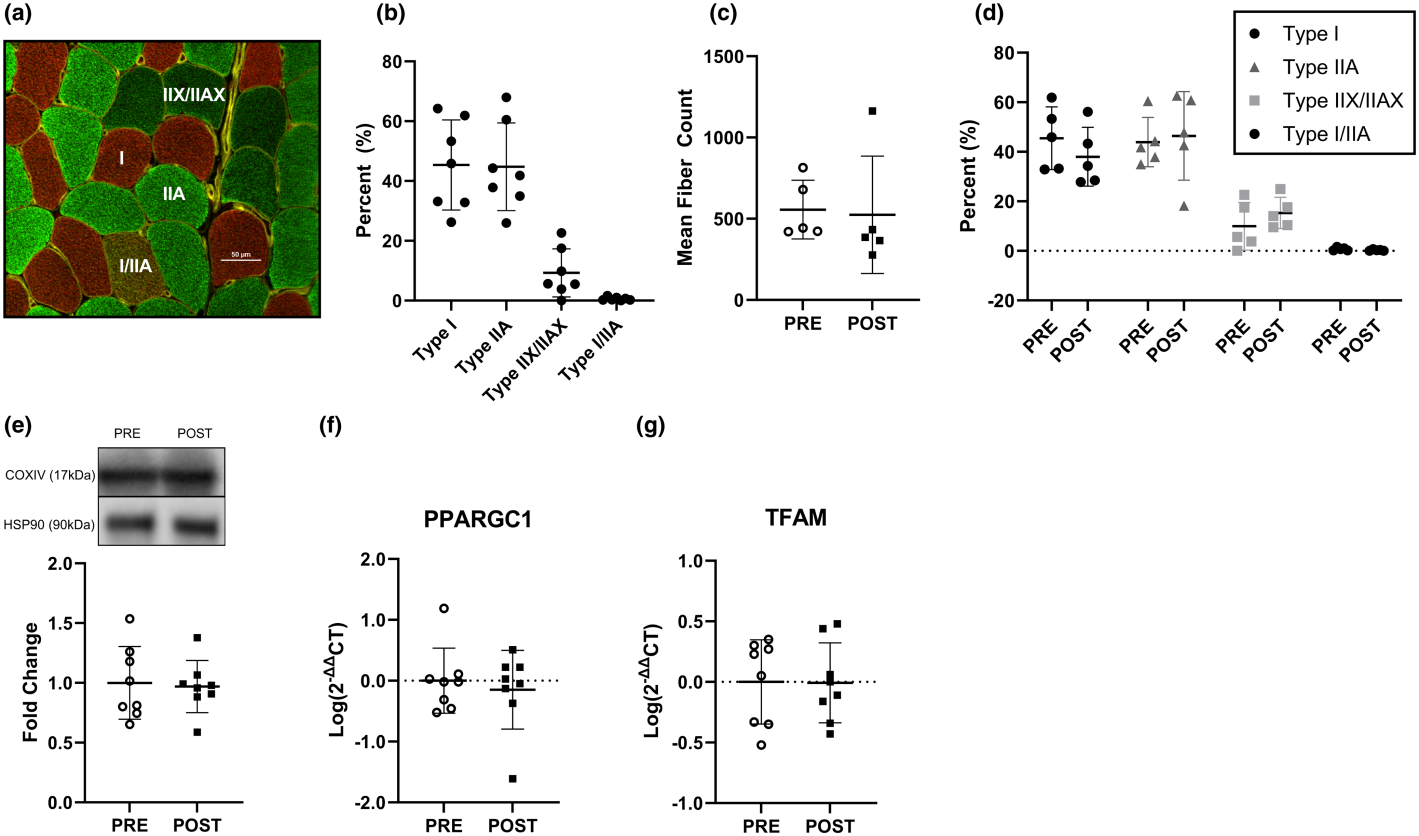
Myofiber type and mitochondrial indices. (a) Representative image of observed myofiber isoforms. Red = Type I, green = IIA, dark green = IIX/IIAX, and red/green = I/IIA. (b) Myofiber type proportion PRE (fibers analyzed = 586 ± 157, *n* = 7). (c) Mean number of myofibers analyzed at PRE and POST (*n* = 5). (d) Myofiber type distribution PRE and POST (*n* = 5). (e) COXIV skeletal muscle protein signaling at PRE and POST timepoints (*n* = 8) (f) PPARGC1 gene expression at PRE and POST timepoints (*n* = 8). (g) TFAM gene expression at PRE and POST timepoints (*n* = 8). All data are represented as mean ± SD. Statistical test = paired *t*‐test. Blot images with the target protein and the molecular weight where membranes were cut for all participants can be found in Figure S3.

Staining for myofiber type (Figure 5a) showed equal proportions of Type I and IIA fibers at baseline (~45% of each myofiber type) and expectedly lower proportions of Type IIX/IIAX and I/IIA (Figure 5b). There was no difference in the number of fibers analyzed or fiber type at POST compared with PRE (Figure 5c,d). There was no difference in COXIV protein content (Figure 5e), or the expression of PPARGC1 and TFAM (Figure 5f,g) from PRE to POST.

**FIGURE 6:**
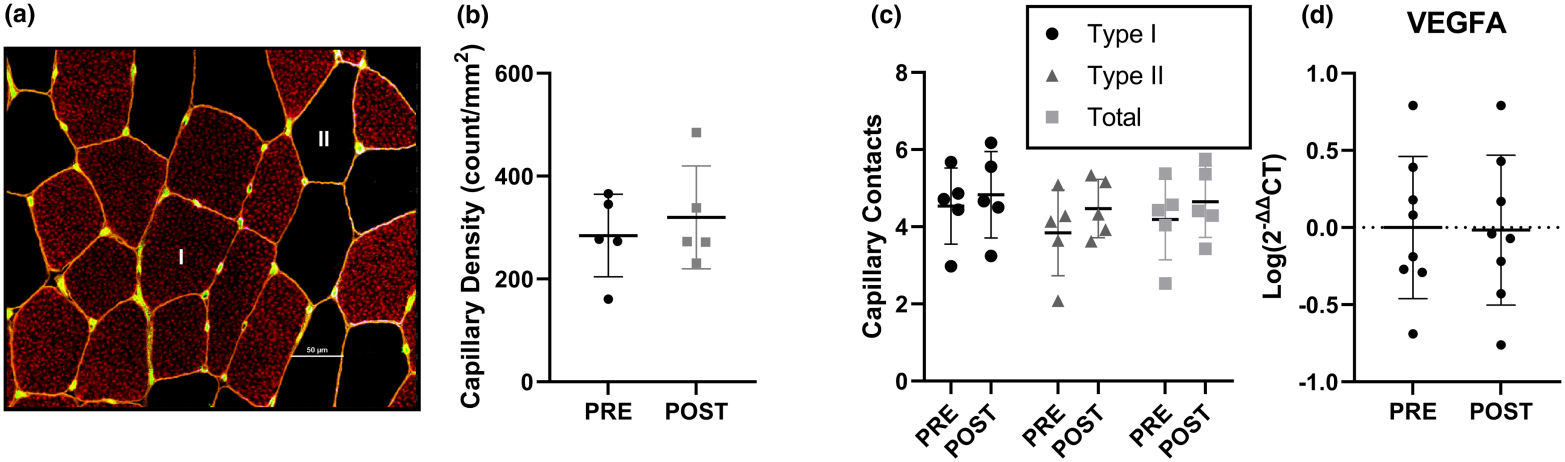
Capillary content and angiogenic gene expression. (a) Representative image of capillary stain. Red = Type I fibers, Black = Type II fibers, green = capillaries. (b) Capillary density PRE and POST (*n* = 5). (c) Capillary contacts by Type I, Type II, and total fibers PRE and POST (*n* = 5). (d) VEGFA skeletal muscle gene expression at PRE and POST timepoints (*n* = 8). All data are represented as mean ± SD. Statistical test = paired T‐test.

Capillary density (Figure 6a) was not different at POST compared with PRE (Figure 6b). Capillary contact with myofibers was not different between PRE to POST (Figure 6c). There was no difference in the expression of VEGFA (Figure 6d) from PRE to POST.

**FIGURE 7:**
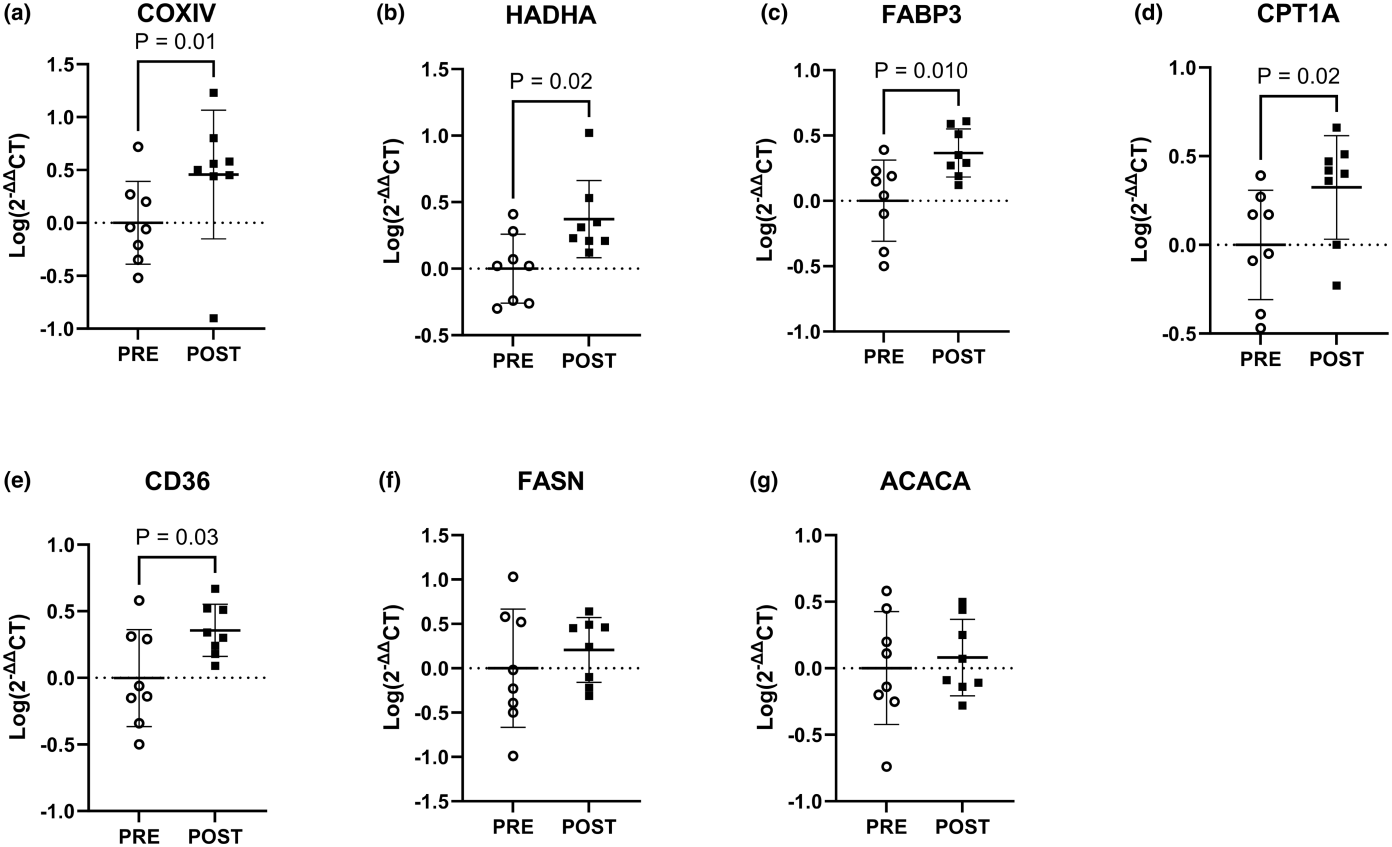
Skeletal muscle gene expression at PRE and POST timepoints. (a) COXIV. (b) HADHA. (c) FABP3. (d) CPT1A. (e) CD36. (f) FASN. (g) ACACA. Data expressed as Log(2^−ΔΔ^CT) relative to PRE. *n* = 8 males. Statistical test = paired *t*‐test.

Expression of COXIV, HADHA, FABP3, CPT1A, and CD36 increased from PRE to POST (*p <* 0.05; Figure 7a–e). There were no differences in the expression of FASN and ACACA from PRE to POST (Figure 7f,g).

## 4 DISCUSSION

This study investigated whether treatment with exogenous EPO could mitigate declines in hematological responses, oxygen carrying capacity, and performance typically experienced by individuals during prolonged periods of strenuous physical activity and overreaching. The major findings from this study were that exogenous EPO not only prevented such declines but produced increases in hematological parameters and improved aerobic performance during 28 days of strenuous exercise training. Increased oxygen carrying capacity resulted in increased V̇O_2_peak, necessitating an increase in the speed during load carriage exercise to match relative intensity (55% V̇O_2_peak) at PRE and POST EPO trials. As a result, energy expenditures were higher at POST than PRE. Those higher energy expenditures during load carriage exercise were supported by higher rates of whole‐body fat oxidation, with no differences in carbohydrate oxidation compared to PRE. Increases in whole‐body fat oxidation may, in part, have been driven by an upregulation in skeletal muscle transcription of markers of mitochondrial activity and fatty acid uptake, transport, and oxidation. These data indicate that exogenous EPO is an effective intervention for sustaining performance for persons who must engage in prolonged (e.g., several weeks) strenuous physical activity with inadequate rest and recovery time.

Enhanced hematological adaptations and aerobic performance during 28 days of EPO treatment in the current study are consistent with previous work (Breenfeldt Andersen et al., 2023; Robach et al., 2009). However, unlike past studies, which assessed the effectiveness of EPO as an ergogenic aid under less extreme conditions, the current design used a model of arduous exercise training that has previously been reported to reduce Hgb and Hct values (Epstein et al., 2018; Hennigar, McClung, et al., 2020; McClung et al., 2013). In the current study, there was no change in Hgb, Hct, and TT performance during the first 2 weeks of the study, suggesting a potential protective effect of EPO. This was followed by an increased oxygen carrying capacity and aerobic performance above PRE values by study Day 21. Effectiveness of EPO on these outcome measures is likely the result of its ability to prompt erythropoiesis and inhibit hepcidin activity (Ashby et al., 2010; Elliott et al., 2008; Lundby & Olsen, 2011; Robach et al., 2009, 2013). EPO increases the number of red blood cells, but requires iron to do so optimally (Tarng et al., 1999). This study supplied sufficient iron, well above the RDA for men (Institute of Medicine Panel on M., 2001), to support hematological function. Sustained military operations pose a unique challenge as they promote inflammation and reduce iron absorption (Hennigar, McClung, et al., 2020). Specifically, our laboratory (Hennigar, McClung, et al., 2020) has previously reported that following a 3‐day simulated military operation where exercise‐induced energy expenditures were ~2000 kcal/day, elevations in IL‐6 and hepcidin resulted in a ~50% reduction in iron absorption, and Hgb and Hct levels decreased, despite participants consuming 22 mg/day iron. EPO concentrations in the current study minimize the exercise induced hepcidin response likely facilitating greater iron absorption and availability. This is supported by studies reporting EPO, in as little as 24 h, reduces hepcidin concentrations (Ashby et al., 2010; Robach et al., 2009, 2013). EPO appears to regulate hepcidin through enhanced secretion of erythroid factors as a byproduct of erythropoiesis opposed to acting upon hepcidin directly (Wang et al., 2017). Therefore, these data demonstrate that EPO drives erythropoiesis despite 28 days of strenuous exercise training, which may be due to reduced hepcidin allowing for the utilization of dietary iron.

To account for increases in V̇O_2_peak following 28 days of EPO treatment and strenuous exercise, the absolute workload during load carriage exercise was increased at POST from PRE, so as to match relative (55% V̇O_2_peak) intensities. Interestingly, the resulting increased exercise‐induced energy expenditures during load carriage exercise at POST were supported by increased fat oxidation, with no changes in carbohydrate oxidation. Increased fat oxidation following EPO administration in the current study agrees with previous studies (Caillaud et al., 2015; Christensen et al., 2012; Larsen et al., 2021). Increased fat oxidation does not appear to be the result of muscle fiber type shifts or increases mitochondrial biogenesis, as there were no differences in skeletal muscle myofiber type distribution or gene expression of PPARGC1 and TFAM following 28 days of EPO treatment and strenuous exercise training. These results are in agreement with previous work reporting no difference in fiber type distribution (Larsen et al., 2014) or PPARGC1 gene expression (Christensen et al., 2013) following 10 weeks of EPO administration with or without moderate exercise training. While EPO does not appear to alter mitochondrial biogenesis, previous studies have suggested that acute (Larsen et al., 2021) and sustained (Guadalupe‐Grau et al., 2015) increases in fat oxidation may be driven by improved mitochondrial respiratory capacity. Results from the current study indicate that same adaptation may account for the increased transcriptional regulation of mitochondrial respiration (HADHA and COXIV), as well as fatty acid uptake (CD36), binding (FABP), and transport (CPT1a) following the EPO treatment and prolonged strenuous exercise. As the oxidative capacity varies within muscle expressing similar myosin isoforms (Ørtenblad et al., 2018), it is likely that EPO in combination with prolonged strenuous exercise elicits muscle adaptations facilitating increased whole‐body fat oxidation by increasing efficiency of mitochondria to oxidize fatty acids, independent of shifts in fiber type.

Despite increases in total energy expenditure during load carriage exercise, there was no change in carbohydrate oxidation following 28 days of EPO with prolonged strenuous exercise training in the current study. This lack of effect of EPO on carbohydrate oxidation conflicts with previous work (Caillaud et al., 2015) which reported that following 28 days of EPO (50 IU/kg 3 × per week) injections carbohydrate oxidation was lower during a bout of cycle ergometry at 75% V̇O_2_max in aerobically trained males. Discordant results between studies may be attributed to differences in exercise intensity (55% vs. 75% V̇O_2_max), with the lower intensity in the current study masking the carbohydrate sparing effects that may have been observed following EPO use at higher exercise intensities. Corroborating the lack of effect on carbohydrate oxidation, the current study also observed no changes in glucose R_a_ and R_d_ from PRE to POST, suggesting that hepatic glycogenolysis and plasma glucose uptake during aerobic exercise remained the same. Though glucose R_a_ and R_d_ were not different, MCR increased from PRE to POST EPO, indicating an increase insulin sensitivity (van Dijk et al., 2013). EPO has been reported to correct insulin resistance in patients on hemodialysis (Mak, 1998; Spaia et al., 2000), but studies in healthy human volunteers have shown no effect of a single dose (Christensen et al., 2012) or 10 weeks (Christensen et al., 2013) of EPO injections on insulin sensitivity. However, insulin sensitivity is enhanced with aerobic exercise training (Christensen et al., 2013). As such, the addition of exercise training to EPO in the current study may have contributed to increased MCR at POST compared with PRE.

There was no effect of 28 days of EPO during strenuous exercise training on muscle capillarization or VEGFA expression in the current study. These finding suggests that angiogenesis did not contribute to the observed enhancements in aerobic performance. These findings agree with Guadalupe‐Grau et al. (2015). who reported no increase in capillary density per muscle fiber or VEGF protein content following 8 weeks of EPO administration in physically active males. Furthermore, while Larsen et al. (2014). reported a 14% increase in capillary density per muscle fiber following 10 weeks of EPO injection combined with moderate exercise training, these findings were not different than exercise training alone. The absence of an increase in capillarization in the current study may either result from the shorter duration (4 vs. 10 weeks), or overreaching blunting angiogenesis (Poffe et al., 2023). Regardless, the lack of difference in muscle capillarization in the current and past studies (Guadalupe‐Grau et al., 2015; Larsen et al., 2014) suggests angiogenesis is unaffected by erythropoiesis, and increased in blood oxygen carrying capacity is likely the primary driver for performance improvements with EPO.

While this study provides novel insight into the impact of EPO administration during prolonged strenuous exercise training, there are limitations. The largest limitation to this study is the lack of a placebo control group. The initial intent of this work was to include a placebo group, however, due to testing restrictions related to the Covid pandemic it was not feasible to execute this study as a parallel placebo‐controlled design. Not having a placebo group precludes the current work from isolating the impact of EPO versus the high level of physical exertion during the 28‐day protocol on outcome measures. Additionally, while females were not excluded from participating, only males consented and were enrolled into the study. As sex‐based differences may exist in response to EPO between males and females (O'Bryan et al., 2022; Zhang et al., 2017), further investigation with female participants is warranted.

In conclusion, results from this study indicate that EPO injections throughout 28 days of strenuous exercise training sustains and enhances hematological status and physical performance. Additionally, 28 days of EPO administration combined with strenuous exercise training resulted in higher rates of whole‐body fat oxidation to support increased exercise‐induced energy expenditures during load carriage exercise matched for relative intensities from PRE. Increases in whole‐body fat oxidation were potentially driven by upregulated transcription of markers of mitochondrial activity and fatty acid uptake, transport, and oxidation within skeletal muscle. These data indicate that EPO can be a safe and effective intervention during strenuous exercise training to increase oxygen carrying capacity, whole‐body and skeletal muscle fat oxidation, and aerobic performance.

## AUTHOR CONTRIBUTIONS

Lee M. Margolis, Stefan M. Pasiakos, James P. McClung, and Benjamin J. Ryan designed research. Devin J. Drummer, Julie L. McNiff, Emily E. Howard, Jess A. Gwin, Christopher T. Carrigan, Nancy E. Murphy, Marques A. Wilson, Julia Michalak, Benjamin J. Ryan, and Lee M. Margolis performed research. Devin J. Drummer and Lee M. Margolis analyzed data; Devin J. Drummer and Lee M. Margolis interpreted results. Devin J. Drummer and Lee M. Margolis prepared tables and figures. Devin J. Drummer and Lee M. Margolis drafted manuscript. Devin J. Drummer, Julie L. McNiff, Emily E. Howard, Jess A. Gwin, Christopher T. Carrigan, Nancy E. Murphy, Marques A. Wilson, Julia Michalak, Benjamin J. Ryan, Stefan M. Pasiakos, James P. McClung, and Lee M. Margolis approved final version. All authors have read and agreed to the published version of the manuscript.

## FUNDING INFORMATION

This material is based on work supported by the Military Operational Medicine Research Program and appointments to the U.S. Army Research Institute of Environmental Medicine administered by the Oak Ridge Institute for Science and Education (to D.J.D., J.L.M, and J.M.) through an interagency agreement between the U.S. Department of Energy and the U.S. Medical Research and Development Command. The funders of the study had no role in the study design, data collection, data analysis, data interpretation, or writing of the manuscript.

## CONFLICT OF INTEREST STATEMENT

The authors have nothing to disclose.

## ETHICS STATEMENT

The investigators adhered to the policies for protection of human subjects as prescribed in Army Regulation 70–25, and the research was conducted in adherence with the provisions of 32 CFR part 219. The opinions or assertions contained herein are the private views of the authors and are not to be construed as official or as reflecting the views of the Army or the Department of Defense. Any citations of commercial organizations and trade names in this report do not constitute an official Department of the Army endorsement of approval of the products.

## Supporting information


Table S1.

Figure S1.

Figure S2.

Figure S3.

## Data Availability

Data are available upon request from the corresponding author, Lee Margolis, lee.m.margolis.civ@health.mil
