# Supplementary material for: Exogenous erythropoietin increases hematological status, fat oxidation, and aerobic performance in males following prolonged strenuous training
Source: Physiol Rep. 2024 May 16;12(10):e16038. doi: 10.14814/phy2.16038 (PMC11099744; doi:10.14814/phy2.16038)
Supplement: Supplementary file 1 — Table S1. [file PHY2-12-e16038-s001.docx]

|  | **Total Study** | **Study Exercise** | **CHO Infusion** | **Time Trial** | **Rest** |
| --- | --- | --- | --- | --- | --- |
| **Kcal/d** | 3161.78 ± 265.11 | 3733.29 ± 230.35 | 3333.73 ± 435.62 | 2846.80 ± 311.09 | 2387.68 ± 283.96 |
| **CHO g/d** | 440.40 ± 36.73 | 518.92 ± 32.85 | 470.15 ± 62.48 | 394.68 ± 42.34 | 334.66 ± 38.26 |
| **PRO g/d** | 124.15 ± 10.83 | 146.56 ± 8.73 | 127.70 ± 20.11 | 110.51 ± 12.52 | 94.76 ± 12.61 |
| **FAT g/d** | 105.84 ± 9.08 | 124.70 ± 7.76 | 110.01 ± 15.11 | 96.75 ± 11.29 | 79.78 ± 9.81 |
| **Kcal/kg/d** | 40.19 ± 1.72 | 47.55 ± 2.67 | 42.21 ± 2.47 | 36.10 ± 1.18 | 30.26 ± 1.35 |
| **CHO g/kg/d** | 5.60 ± 0.24 | 6.61 ± 0.37 | 5.95 ± 0.35 | 5.00 ± 0.14 | 4.24 ± 0.18 |
| **PRO g/kg/d** | 2.21 ± 1.82 | 2.63 ± 2.22 | 2.21 ± 1.67 | 1.94 ± 1.53 | 1.67 ± 1.34 |
| **FAT g/kg/d** | 2.78 ± 4.10 | 3.33 ± 4.99 | 2.89 ± 4.27 | 2.45 ± 3.47 | 2.05 ± 2.97 |
| **CHO %** | 55.72 ± 0.11 | 55.59 ± 0.11 | 56.40 ± 1.22 | 55.47 ± 0.39 | 56.09 ± 0.54 |
| **PRO %** | 15.70 ± 0.15 | 15.71 ± 0.09 | 15.30 ± 0.99 | 15.52 ± 0.29 | 15.85 ± 0.39 |
| **FAT %** | 30.12 ± 0.28 | 30.06 ± 0.21 | 29.71 ± 1.48 | 30.57 ± 0.29 | 30.06 ± 0.65 |
| **Iron (mg)** | 39.48 ± 4.40 | 42.45 ± 4.63 | 36.31 ± 3.51 | 36.80 ± 3.65 | 36.40 ± 4.55 |
| **Fiber (g)** | 26.59 ± 3.22 | 29.72 ± 3.53 | 33.42 ± 5.67 | 24.69 ± 2.28 | 21.85 ± 3.33 |
| **Sat Fat (g)** | 39.67 ± 3.39 | 47.41 ± 2.88 | 36.10 ± 6.09 | 36.19 ± 4.35 | 29.40 ± 3.77 |
| **Vit C (mg)** | 215.71 ± 36.37 | 210.62 ± 37.78 | 211.59 ± 20.71 | 253.89 ± 54.58 | 205.16 ± 29.55 |
| **Calcium (mg)** | 1480.29 ± 87.39 | 1738.83 ± 77.67 | 1388.25 ± 170.06 | 1298.61 ± 125.42 | 1166.68 ± 110.83 |
| **Caffeine (mg)** | 0.63 ± 0.92 | 1.13 ± 1.80 | 0.00 ± 0.00 | 0.45 ± 1.27 | 0.00 ± 0.00 |
| CHO = Carbohydrate, PRO = Protein; Mean ± SD; n = 8 | | | | | |

**Supplementary Table 1. Energy and Nutrient Status by Daily Activity Requirements**
