# Supplementary material for: Exogenous erythropoietin increases hematological status, fat oxidation, and aerobic performance in males following prolonged strenuous training
Source: Physiol Rep. 2024 May 16;12(10):e16038. doi: 10.14814/phy2.16038 (PMC11099744; doi:10.14814/phy2.16038)
Supplement: Supplementary file 2 — Figure S1. [file PHY2-12-e16038-s004.pdf]

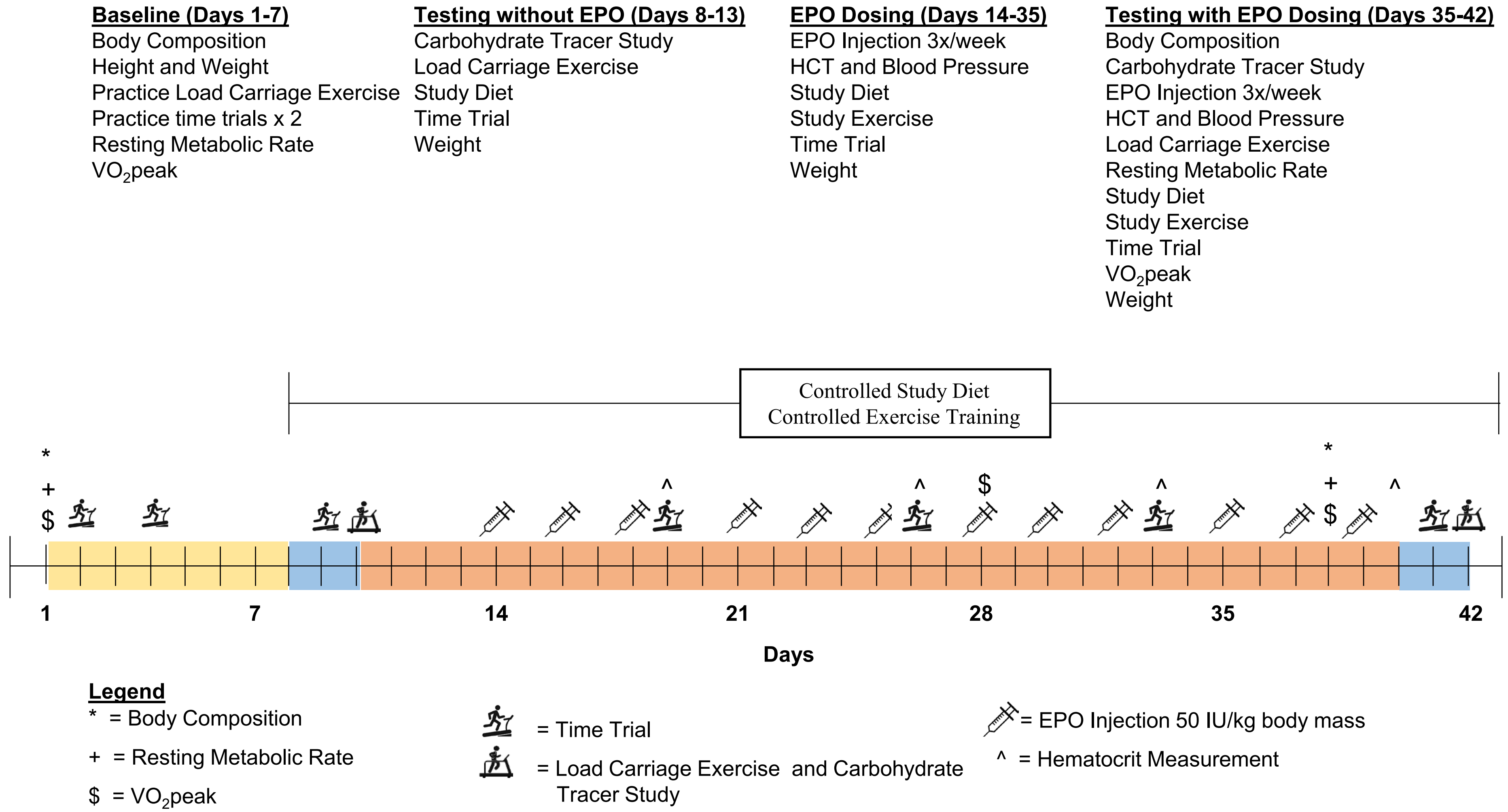
Baseline (Days 1-7)
Body Composition
Height and Weight
Practice Load Carriage Exercise
Practice time trials x 2
Resting Metabolic Rate
VO2peak
Testing without EPO (Days 8-13)
Carbohydrate Tracer Study
Load Carriage Exercise
Study Diet
Time Trial
Weight
EPO Dosing (Days 14-35)
EPO Injection 3x/week
HCT and Blood Pressure
Study Diet
Study Exercise
Time Trial
Weight
Testing with EPO Dosing (Days 35-42)
Body Composition
Carbohydrate Tracer Study
EPO Injection 3x/week
HCT and Blood Pressure
Load Carriage Exercise
Resting Metabolic Rate
Study Diet
Study Exercise
Time Trial
VO2peak
Weight
Controlled Study Diet
Controlled Exercise Training
1
7
14
21
28
35
42
Days
Legend
* = Body Composition
+ = Resting Metabolic Rate
$ = VO2peak
= Time Trial
= Load Carriage Exercise and Carbohydrate Tracer Study
= EPO Injection 50 IU/kg body mass
^ = Hematocrit Measurement
