# Supplementary material for: Exogenous erythropoietin increases hematological status, fat oxidation, and aerobic performance in males following prolonged strenuous training
Source: Physiol Rep. 2024 May 16;12(10):e16038. doi: 10.14814/phy2.16038 (PMC11099744; doi:10.14814/phy2.16038)
Supplement: Supplementary file 3 — Figure S2. [file PHY2-12-e16038-s003.pdf]

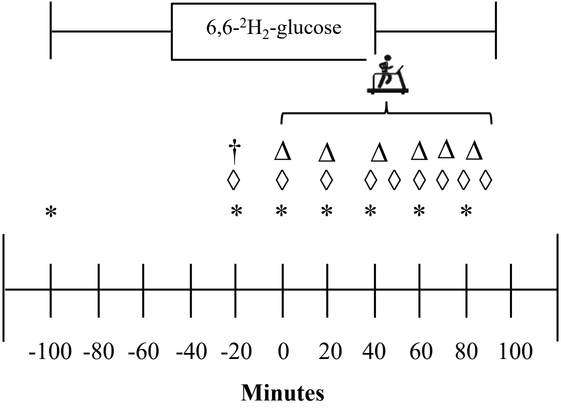

Figure Legend:

= Steady-State load Carriage Exercise (55 ± 5% VO_2peak)

* = Blood Sampling

Δ = VO_2/VCO_2 and Heart Rate

◊ = Breath Sampling

† = Muscle biopsy
