# Supplementary figures and images for: Exogenous erythropoietin increases hematological status, fat oxidation, and aerobic performance in males following prolonged strenuous training
Source: Physiol Rep. 2024 May 16;12(10):e16038. doi: 10.14814/phy2.16038 (PMC11099744; doi:10.14814/phy2.16038)

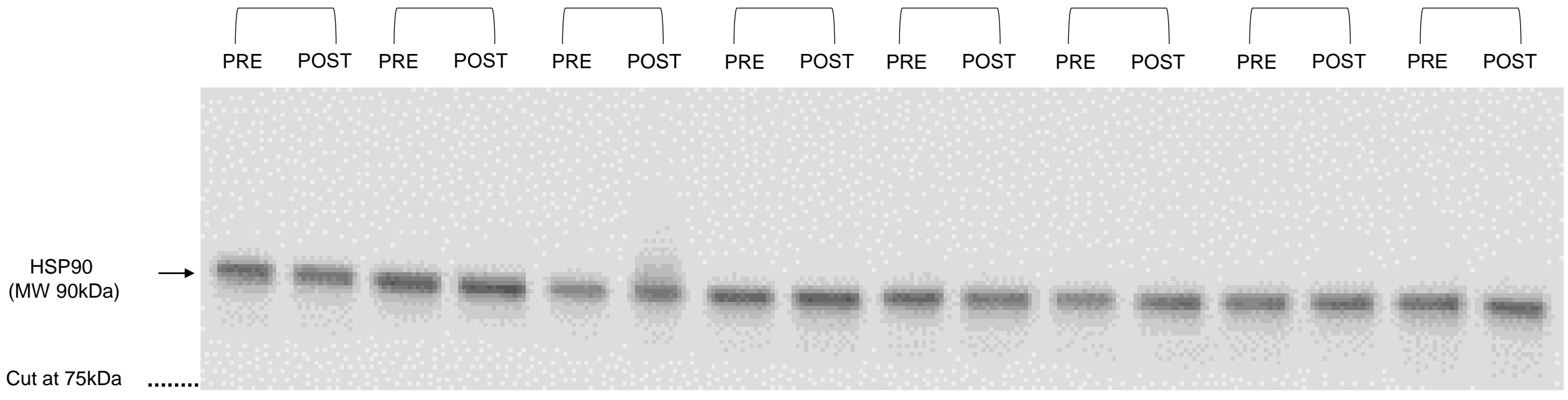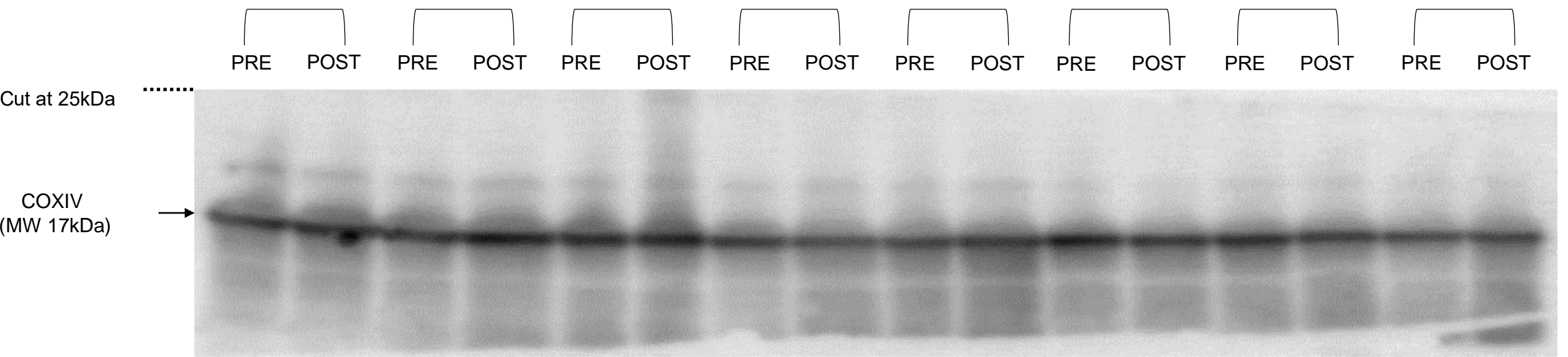

Supplement: Supplementary file 4 — Figure S3. [file PHY2-12-e16038-s002.pdf]
